# MultiNEP: a multi-omics network enhancement framework for prioritizing disease genes and metabolites simultaneously

**DOI:** 10.1093/bioinformatics/btad333

**Published:** 2023-05-22

**Authors:** Zhuoran Xu, Luigi Marchionni, Shuang Wang

**Affiliations:** Department of Pathology and Laboratory Medicine, Weill Cornell Medicine, New York, NY 10065, United States; Department of Pathology and Laboratory Medicine, Weill Cornell Medicine, New York, NY 10065, United States; Department of Biostatistics, Columbia University, New York, NY 10032, United States

## Abstract

**Motivation:**

Many studies have successfully used network information to prioritize candidate omics profiles associated with diseases. The metabolome, as the link between genotypes and phenotypes, has accumulated growing attention. Using a ”multi-omics” network constructed with a gene–gene network, a metabolite–metabolite network, and a gene–metabolite network to simultaneously prioritize candidate disease-associated metabolites and gene expressions could further utilize gene–metabolite interactions that are not used when prioritizing them separately. However, the number of metabolites is usually 100 times fewer than that of genes. Without accounting for this imbalance issue, we cannot effectively use gene–metabolite interactions when simultaneously prioritizing disease-associated metabolites and genes.

**Results:**

Here, we developed a *Multi*-omics *N*etwork *E*nhancement *P*rioritization (MultiNEP) framework with a weighting scheme to reweight contributions of different sub-networks in a multi-omics network to effectively prioritize candidate disease-associated metabolites and genes simultaneously. In simulation studies, MultiNEP outperforms competing methods that do not address network imbalances and identifies more true signal genes and metabolites simultaneously when we down-weight relative contributions of the gene–gene network and up-weight that of the metabolite–metabolite network to the gene–metabolite network. Applications to two human cancer cohorts show that MultiNEP prioritizes more cancer-related genes by effectively using both within- and between-omics interactions after handling network imbalance.

**Availability and implementation:**

The developed MultiNEP framework is implemented in an R package and available at: https://github.com/Karenxzr/MultiNep

## 1 Introduction

Combining multiple types of omics profiles across different omics layers enables a more comprehensive description from genotypes to phenotypes and can better understand biological processes (Ideker *et al.* 2001). Metabolomics accumulated growing attention recently due to its critical position in biological pathways (Fiehn 2002). As the end products of gene expressions and protein activities, metabolite abundances reflect upstream genetic alterations and environmental changes. Cross-talks between metabolites and other types of omics profiles provide additional insights in disease mechanisms. For instance, differential gene expressions could trigger metabolism reprogramming that is required for cell proliferation and transformation in cancers (Yoshida 2015). In return, metabolite alterations lead to aberrant gene expressions through modifying epigenetic processes and interfering with transcriptional regulators (Fu *et al.* 2021). Therefore, jointly analyzing metabolomics and other omics provides opportunities to better understand disease mechanisms and to identify novel therapeutic targets. Metabolites have been associated with diseases, such as diabetes (Arneth *et al.* 2019), chronic kidney disease (Lee *et al.* 2020), and cancers (Röhnisch *et al.* 2020, Bendinelli *et al.* 2021). Multiple databases were also developed to facilitate functional studies of metabolites (Sud *et al.* 2016, Brunk *et al.* 2018).

Network-assisted methods were developed to use biological networks with additional interaction information among genes, metabolites, etc., to help prioritize candidate disease-associated omics profiles (Wang *et al.* 2019), or to identify disease-associated sub-networks (Ruan *et al.* 2016), such as using gene–gene networks [undirected protein-protein interaction (PPI) networks (Köhler *et al.* 2008) or directed gene regulatory networks (GRN) (Dimitrakopoulos *et al.* 2018)]. With network information, omics profiles with weak disease association signals but interact with features having strong disease association signals may be re-prioritized higher for next step functional analysis. Köhler *et al.* (2008) developed GeneWanderer to prioritize disease genes using a PPI network through a diffusion process. We recently developed DiSNEP that enhances a general PPI into a disease-specific network using disease gene expression profiles which achieves better prioritization results (Ruan and Wang 2021). Using a gene–gene network, these methods were applied to other omics profiles that can be mapped to genes. For instance, Dimitrakopoulos *et al.* (2018) developed NetICS to prioritize cancer genes by combining gene-level signals from various omics types, including genomic, epigenomic, transcriptomic, and proteomic profiles, into overall gene alteration scores followed by a diffusion process on a directed GRN. Yao *et al.* (2015) developed MetPriCNet and used a composite network to update known disease-metabolite lists in the Human Metabolome Database (HMDB). Here a composite network includes a gene–gene (g–g) PPI network from the STRING database (Jensen *et al.* 2009), a metabolite–metabolite (m–m), and a gene–metabolite (g–m) network from the STITCH database (Kuhn *et al.* 2008), a phenotype–phenotype network from the MimMiner database (van Driel *et al.* 2006) and a gene–phenotype association network from the OMIM database (Amberger and Hamosh 2017). However, MetPriCNet does not use disease omics profiles to prioritize candidate disease-associated metabolites.

No methods exist to prioritize genes and metabolites simultaneously using multi-omics networks. To simultaneously prioritize genes and metabolites, in addition to g–g interactions within a g–g network or m–m interactions within an m–m network, we can also use additional g–m interactions within a g–m network to help to prioritize signal genes, and signal metabolites compare to prioritizing them separately. A simple extension of current network-assisted methods could be to construct a multi-omics network having a g–g network, an m–m network, and a g–m network. However, there are usually a few hundreds annotated detectable metabolites in a cohort (Krassowski *et al.* 2020), with about 5400 known compounds in the reference library of Metabolon’s metabolomics platform (https://www.metabolon.com/technology-knowledgebase/). While there are over 20K genes and over 850K methylation sites in the Illumina MethylationEPIC BeadChip. It is analytically challenging when using network-assisted methods to simultaneously prioritize candidate disease-associated metabolites and gene expressions/DNA methylations. We need to account for this imbalance issue. Otherwise, g–g interactions can easily dominate g–m interactions when prioritizing genes, and g–m interactions can easily dominate m–m interactions when prioritizing metabolites using a multi-omics network. To overcome these challenges, we developed a *Multi*-omics *N*etwork *E*nhancement *P*rioritization framework, MultiNEP, to prioritize candidate disease-associated genes and metabolites simultaneously. MultiNEP uses a disease-specific multi-omics network with a weighting scheme to reweight the relative contributions of different sub-networks to handle the imbalance issue. Similar to our previous work DiSNEP (Ruan and Wang 2021), MultiNEP enhances a general multi-omics network using disease omics profiles through a diffusion process, during which two weighting parameters are introduced to control for relative contributions of sub-networks. We conducted simulation studies to (i) examine the effects of the weighting parameters and (ii) evaluate the performance of MultiNEP to prioritize candidate disease-associated genes and metabolites simultaneously compared with competing methods that either does not handle network imbalance or prioritize different types of omic profiles separately. MultiNEP outperforms competing methods and prioritizes more disease-associated genes and metabolites simultaneously using both within- and between-omics interactions in all simulation scenarios with appropriate values of the weighting parameters. In real data applications to prioritize candidate cancer-associated genes and metabolites using data from the Dana-Farber/Harvard Cancer Center (DF/HCC) Prostate Cancer Cohort (Xu *et al.* 2022) and the GSE37751 Breast Cancer cohort (Terunuma *et al.* 2014), MultiNEP consistently identifies more disease-related genes according to the DisGeNET (Piñero *et al.* 2017) database.

## 2 Materials and methods

**Figure 1.**
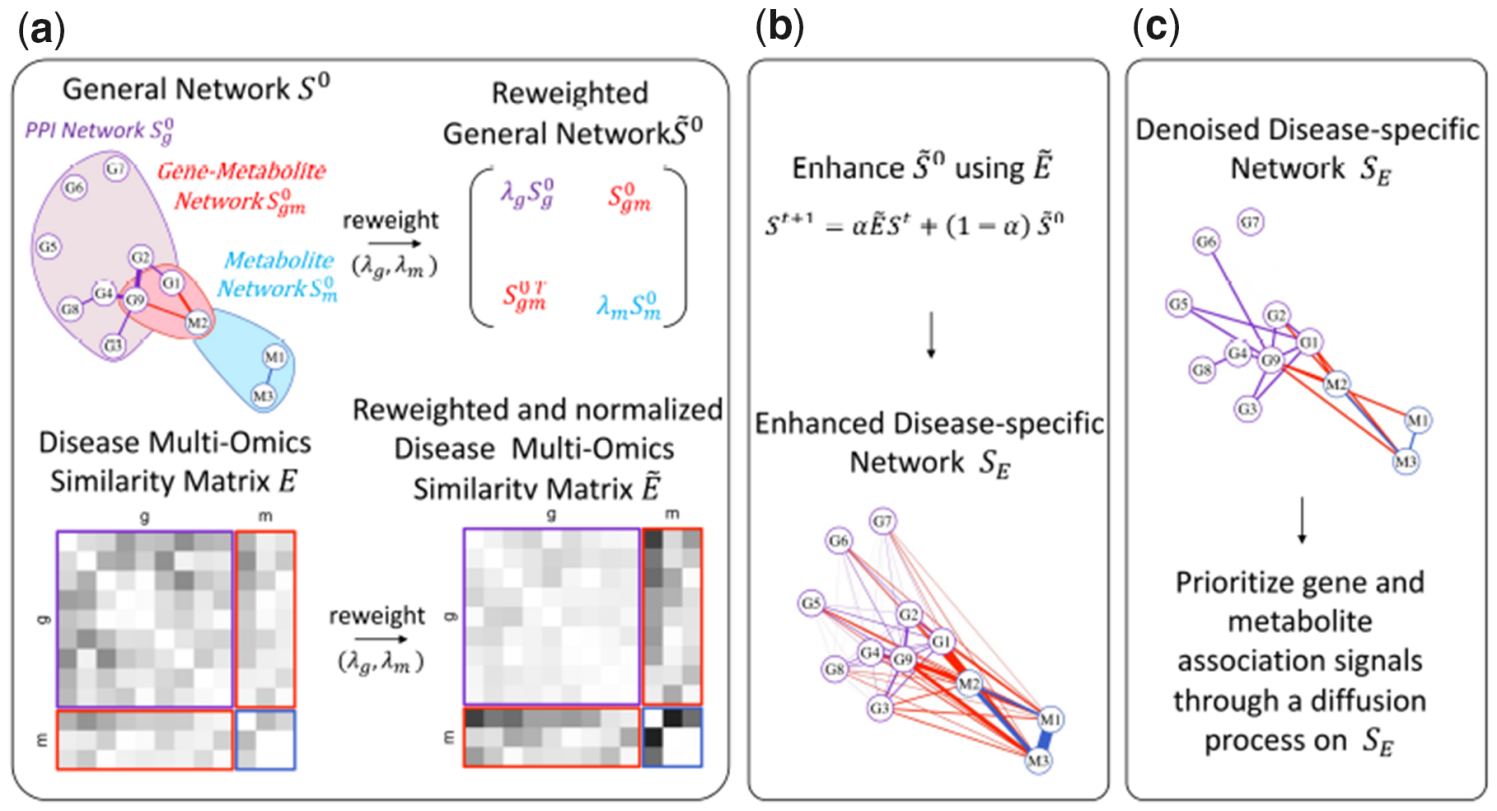
The workflow of MultiNEP.

Figure 1 displays MultiNEP workflow. MultiNEP has three steps. (i) Reweight a general multi-omics network $S^{0}$ and a disease multi-omics similarity matrix *E* into $\tilde{S^{0}}$ and $\tilde{E}$ with the proposed two weighting parameters $\lambda_{g}$ and $\lambda_{m}$. (ii) Use reweighted $\tilde{E}$ to enhance reweighted $\tilde{S^{0}}$ into a disease-specific network $S_{E}$. (iii) Prioritize association scores by diffusing on the enhanced multi-omics network $S_{E}$ to prioritize candidate disease-associated genes and metabolites simultaneously.

### 2.1 Step 1: Reweight a general multi-omics network $S^{0}$ into $\tilde{S^{0}}$ and a disease similarity matrix *E* into $\tilde{E}$

Denote a multi-omics general network as: $S^{0}=\left[\begin{array}{cc} S_{g}^{0} & S_{gm}^{0}\\ S_{gm}^{0T} & S_{m}^{0} \end{array}\right],$ where $S_{g}^{0}$ is the PPI (g–g) network from STRING V11.5 (Jensen *et al.* 2009), $S_{m}^{0}$ and $S_{gm}^{0}$ are the general m–m network and g–m network, respectively, from STITCH V5.0 (Kuhn *et al.* 2008). STRING and STITCH have the same scoring system for pairwise functional interactions. No extra scaling procedures are needed to combine $S_{g}^{0}$, $S_{m}^{0}$ and $S_{gm}^{0}$ into a general multi-omics network $S^{0}$. This $S^{0}$ is an undirected and weighted network with edge weights ranging from 0 to 999, representing the confidence of pairwise functional interactions. Over 95% of edges in $S_{g}^{0}$ and $S_{gm}^{0}$ and about 80% of edges in $S_{m}^{0}$ have 0 weights. There are about 18 000 nodes in $S_{g}^{0}$ and $S_{gm}^{0}$, and about 200 nodes in $S_{m}^{0}$, with each node representing a gene or a metabolite. To account for imbalanced dimensions in gene expressions and metabolites in order to effectively use the multi-omics network to prioritize candidate disease-associated gene expressions and metabolites simultaneously, we introduce two weighting parameters $\lambda_{g}$ and $\lambda_{m}$ to reweight relative contributions of $S_{g}^{0}$ and $S_{m}^{0}$ to $S_{gm}^{0}$ as follows: where $\lambda_{g}$ and $\lambda_{m}$ control the contributions of $S_{g}^{0}$ and $S_{m}^{0}$ relatively to $S_{gm}^{0}$, respectively. A value of $0<\lambda_{g}<1$ decreases the contribution of $S_{g}^{0}$ relatively to $S_{gm}^{0}$. A value of $\lambda_{m}>1$ increases the contribution of $S_{m}^{0}$ relatively to $S_{gm}^{0}$. Because the size of $S_{g}^{0}$ is much larger than that of $S_{gm}^{0}$, we would like to set $\lambda_{g}$ a value much smaller than 1 and $\lambda_{m}$ a value much larger than 1. Note that when setting $\lambda_{g}=\lambda_{m}=1$, it is a direct extension of DiSNEP (Ruan and Wang 2021) by treating $S_{g}^{0}$, $S_{m}^{0}$, and $S_{gm}^{0}$ equally.


$$\tilde{S^{0}}=\left[\begin{array}{cc} \lambda_{g}S_{g}^{0} & S_{gm}^{0}\\ S_{gm}^{0T} & \lambda_{m}S_{m}^{0} \end{array}\right],$$

We enhance the reweighted general network $\tilde{S^{0}}$ into a disease-specific network $S_{E}$ for a disease of interest as we have previously demonstrated its effectiveness in prioritizing candidate disease-associated signals (Ruan and Wang 2021). To do so, we generate a disease multi-omics similarity matrix *E* using disease multi-omics data which reflects real data g–g, m–m, and g–m interactions of the disease, where we use absolute values of pairwise correlations: $E=\left[\begin{array}{cc} E_{g} & E_{gm}\\ E_{gm}^{T} & E_{m} \end{array}\right].$ Here $E_{g}$, $E_{m}$, and $E_{gm}$ are g–g, m–m, and g–m similarity matrices with pairwise correlations. We similarly reweight *E* with $\lambda_{g}$ and $\lambda_{m}$ to control the relative contributions of $E_{g}$ and $E_{m}$ to $E_{gm}$: $\tilde{E}=\left[\begin{array}{cc} \lambda_{g}E_{g} & E_{gm}\\ E_{gm}^{T} & \lambda_{m}E_{m} \end{array}\right].$ We then conduct column normalization on $\tilde{E}$ to have a stochastic matrix to guarantee convergence (Köhler *et al.* 2008). We use the same values of $\lambda_{g}$ and $\lambda_{m}$ to reweight $S^{0}$ into $\tilde{S^{0}}$, and *E* into $\tilde{E}$, because the two matrices have the same imbalance issue with the same number of genes and number of metabolites.

### 2.2 Step 2: Enhance the general network $\tilde{S^{0}}$ into a disease-specific network $S_{E}$ using $\tilde{E}$

With reweighted $\tilde{E}$, we enhance reweighted network $\tilde{S^{0}}$ using random walk with restart method to generate a disease specific multi-omics network which better reflects multi-omics interactions of the disease:


$$S^{t+1}=\alpha \tilde{E}S^{t}+(1-\alpha )\tilde{S^{0}}.$$

Here, α controls the degree of enhancement where α=0 means no enhancement using disease omics data, and *t* is iteration steps. We set α=0.75 as previously indicated (Ruan and Wang 2021), and end iterations when $||S^{t+1}-S^{t}|{|}_{1}<1\times 10^{-6}$ where $S^{t}$ = $\tilde{S^{0}}$ when t=0. We then symmetrize $S^{t}$ as follows: $(S^{t}+S^{tT})/2$ and denoise each sub-networks separately by removing ”noisy edges” where we kept top 5% edges ranked with edge weights in $S_{g}^{t,\mathrm{sym}}$; 30% edges in $S_{m}^{t,\mathrm{sym}}$, and 15% edges in $S_{gm}^{t,\mathrm{sym}}$. For the choices of these numbers, see Supplementary Materials. After denoising, we obtain the final enhanced multi-omics network $S_{E}$, and conduct column normalization on $S_{E}$ to guarantee convergence for signal prioritization.

### 2.3 Step 3: Prioritize association signals

We obtain disease association scores for each gene and metabolite using appropriate statistical tests, e.g. regression-based or others, with higher scores indicating stronger associations. For example, with a case-control design, we can perform a two-sample t-test to compare means in gene expressions or metabolites between cases and controls and obtain *P*-values $p_{g}$ and $p_{m}$, respectively. Association scores for genes and metabolites are $v^{0}=(v_{g}^{0},v_{m}^{0})=(\phi^{-1}(1-p_{g}/2),\phi^{-1}(1-p_{m}/2))$, with ϕ being standard normal cumulative distribution function. We then use the reweighted and disease-specific network $S_{E}$ from Step 2 to prioritize candidate disease-associated gene expressions and metabolites simultaneously by diffusing $v^{0}=(v_{g}^{0},v_{m}^{0})$ on $S_{E}$ and obtain prioritized association signals $v=(v_{g},v_{m})$:


$$v^{t+1}=\beta S_{E}v^{t}+(1-\beta )v^{0}.$$

Here, β controls the degree of enhancement to update $v^{0}$ using $S_{E}$, *t* is iteration steps and we end iterations when $||v^{t+1}-v^{t}|{|}_{1}<1\times 10^{-6}$. We set β=0.75 as suggested in Ruan and Wang (2021). Note that disease multi-omics data is used twice: to enhance a general multi-omics network to a disease-specific network and to generate disease association scores.

## 3 Results

### 3.1 Simulation studies

We conducted simulation studies to investigate the performance of MultiNEP in prioritizing disease-associated genes and metabolites simultaneously. No methods exist that simultaneously prioritize multiple types of omics profiles. We extended GeneWanderer (Köhler *et al.* 2008) and DiSNEP (Ruan and Wang 2021) as competing methods and compared MultiNEP to: (i) General Network that uses a general multi-omics network without enhancing it to a disease-specific network nor reweighting individual sub-networks, and (ii) DiSNEP that uses an enhanced disease-specific multi-omics network without reweighting individual sub-networks (i.e. MultiNEP with $\lambda_{g}=\lambda_{m}=1$). We performed same denoising procedures for networks of all methods.

#### 3.1.1 Evaluation metrics

To evaluate prioritization performance, we used average numbers of true signal genes and metabolites out of top ranked 1–500 prioritized combined features over 100 simulations. We also defined an interaction ratio score (IRS) that measures whether a feature (gene/metabolite) in a network has stronger interactions relative to the average. For example, the IRS of a gene in the g–g network is the ratio of total connections/scores of this gene to the average connections/scores of a gene in this network. IRS is similarly defined for genes/metabolites in all sub-networks.

#### 3.1.2 Obtaining a general multi-omics network

We obtained a general multi-omics network $S^{0}$ from STRING and STITCH databases and trimmed $S^{0}$ to only keep genes and metabolites existing in a disease cohort (Supplementary Table S1). There are 18 009 genes and 203 metabolites in the prostate cancer cohort from the Dana-Farber/Harvard Cancer Center (DF/HCC) (Supplementary Table S2). We downloaded the list of 632 genes associated with prostate cancer from the DisGeNET database V7.0 (Piñero *et al.* 2017) as the known prostate cancer-related genes, the rest 17 377 as noise genes. To the best of our knowledge, there is no disease-associated metabolite database.

In simulation studies, we set a total of 5000 genes and 60 metabolites in a general network $S^{0}$ to mimic the imbalanced multi-omics network observed in real data. Among 5000 genes, 175 were randomly selected from the list of 632 prostate cancer-related genes as true signal genes, and 4825 genes were randomly selected from the rest 17 377 noise genes in $S^{0}$ from the STRING network as noises. For metabolites, we randomly selected 60 metabolites from the 203 metabolites in $S^{0}$, with 12 out of 60 were randomly set as true signal metabolites and the remaining 48 as noises.

#### 3.1.3 Simulating disease multi-omics profiles

We generated omics profiles of 100 cases and 100 controls from multivariate normal distributions N(μ,Σ), $\Sigma_{ij}=\rho_{ij}\sigma_{i}\sigma_{j}$, i,j={1,…,5,060}, where μ is a mean vector, $\rho_{ij}$ is correlation between omics features *i* and *j*, and σ is standard deviation. Parameter values were chosen to mimic real prostate cancer gene expression and metabolite data. Although signal genes/metabolites are expected to be up/down-regulated in cases comparing to controls, we only consider upregulated signals in cases for simplicity and set μ=6.15 for the 175 signal genes and μ=0.15 for the 12 signal metabolites of cancer patients. We set μ=6 for the 4825 noise genes of cancer patients and all 5000 genes of healthy controls, and μ=0 for the 48 noise metabolites of cancer patients and all 60 metabolites of healthy controls. We set σ=2 for gene expressions and σ=2.5 for metabolites. Correlations between signal genes are higher than that between signal genes and noise genes, and correlations between noise genes are smallest (Supplementary Fig. S3). Thus, we set $\rho_{ij}=0.25$ between signal genes, $\rho_{ij}=0.20$ between noise genes, $\rho_{ij}=0.225$ between signal and noise genes, $\rho_{ij}=0.30$ between signal metabolites, $\rho_{ij}=0.15$ between noise metabolites, and $\rho_{ij}=0.225$ between signal and noise metabolites.

To investigate how g–m interactions help prioritize signal genes and metabolites, we considered a series of signal g–m correlations $\rho_{ij}=0.05,0.2,0.35,0.5$, which are 50th, 95th, 99th, and 99.9th percentiles of g–m correlations in the prostate cancer data (Supplementary Fig. S3). With these values, we set the following possible correlations between signal genes and signal metabolites, signal genes and noise metabolites, noise genes and signal metabolites, and noise genes and noise metabolites $\rho_{ij}=\{0.05,0.03,0.03,0.01\}$, $\rho_{ij}=\{0.2,0.105,0.105,0.01\}$, $\rho_{ij}=\{0.35,0.18,0.18,0.01\}$, and $\rho_{ij}=\{0.5,0.225,0.225,0.01\}$. We conducted 100 simulations for each simulation setting and considered different values of the weighting parameters $\lambda_{g}$ and $\lambda_{m}$ of MultiNEP. For MultiNEP and DiSNEP that use disease omics data to enhance a general network into a disease-specific network, we set α and β as 0.75 (Ruan and Wang 2021).

### 3.2 Simulation results

#### 3.2.1 Compare prioritization results using a multi-omics network versus a single-omics network

The rationales underlying the proposed MultiNEP have two folds: (i) to use g–m interactions in a multi-omics network to simultaneously prioritize gene expressions and metabolites compared to using a single-omics network to prioritize gene expressions and metabolites separately, and (ii) to account for different dimensions of gene expressions and metabolites to better use both between- and within-omics interactions in a multi-omics network to prioritize more true signal genes and metabolites simultaneously.

We compared the extended DiSNEP that starts with a multi-omics network $S^{0}$ to the original DiSNEP that starts with either a g–g network $S_{g}^{0}$ or an m–m network $S_{m}^{0}$ separately. Supplementary Figure S1 shows that the extended DiSNEP that uses additional g–m interactions consistently prioritizes more signal genes and metabolites than that of the original DiSNEP although the improvements are minimum when ignoring the network imbalance issue. We next investigated the effect of the weighting parameters $\lambda_{g}$ and $\lambda_{m}$. We use DiSNEP to denote the extended DiSNEP, i.e. MultiNEP when $\lambda_{g}=\lambda_{m}$ = 1.

#### 3.2.2 Determine optimal values of $\lambda_{g}$ and $\lambda_{m}$

**Figure 2.**
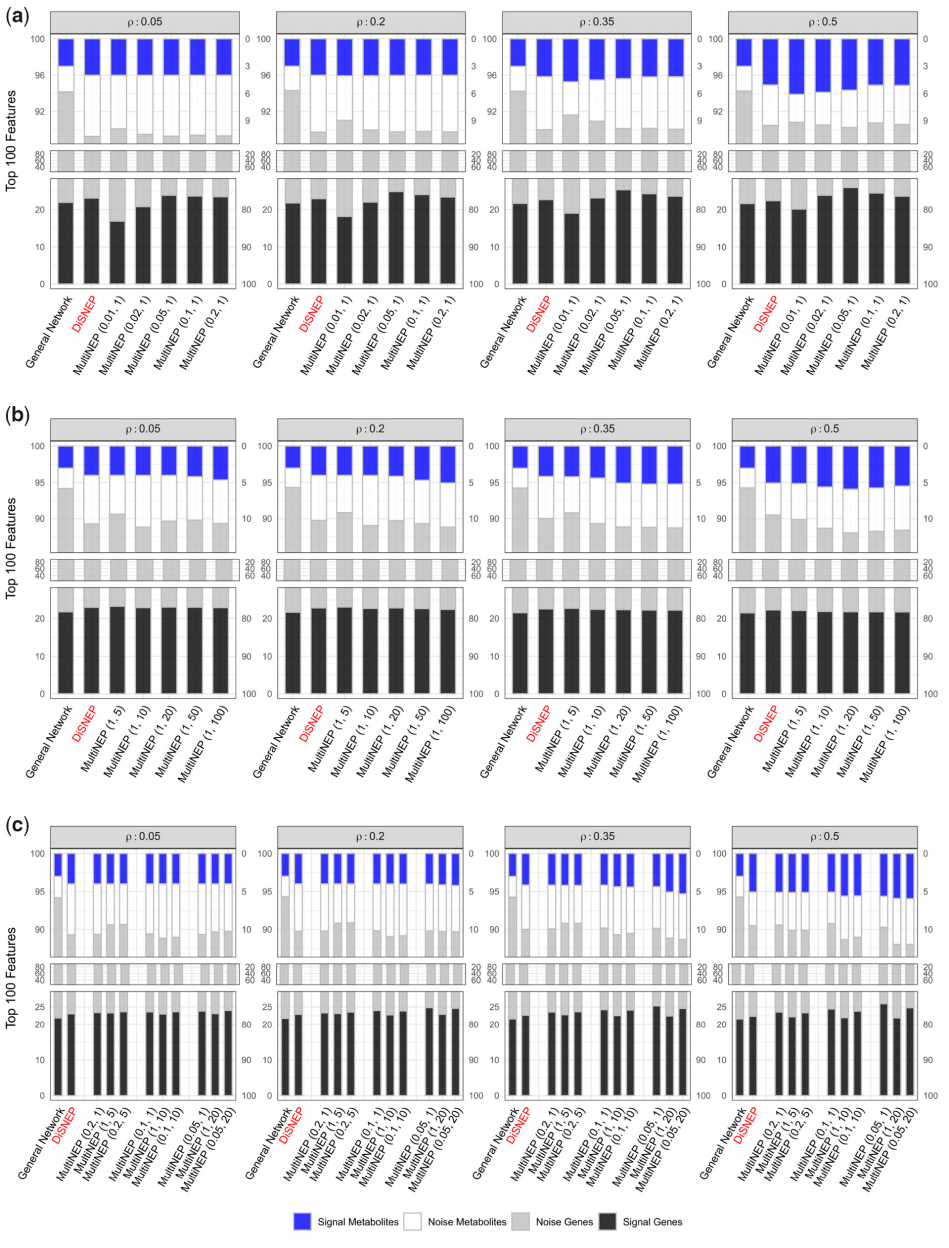
Simulation studies to determine optimal values of $\lambda_{g}$ and $\lambda_{m}$ for MultiNEP. (a) $\lambda_{g}$ = 0.01, 0.02, 0.05, 0.1, 0.2 and $\lambda_{m}$ = 1; (b) $\lambda_{g}$ = 1 and $\lambda_{m}$= 5, 10, 20, 50, 100); (c) $\lambda_{g}$ = 0.2, 0.1, 0.05, 1 and $\lambda_{m}$ = 1, 5, 10, 20 for MultiNEP ($\lambda_{g}$, $\lambda_{m}$). Displayed are average numbers of signal genes, signal metabolites, noise genes, and noise metabolites out of top ranked 100 prioritized combined features across 100 simulations when signal g–m correlations are ρ=0.05,0.2,0.35,0.5.

We conducted simulations to determine optimal values of $\lambda_{g}$ and $\lambda_{m}$. We set $\lambda_{g}$ = 0.01, 0.02, 0.05, 0.1, 0.2 and fixed $\lambda_{m}$ = 1 to study the effect of $\lambda_{g}$, and set $\lambda_{m}$ = 5, 10, 15, 20, 50 and fixed $\lambda_{g}$ = 1 to study the effect of $\lambda_{m}$. Figure 2 displays average numbers of true signal genes (black), true signal metabolites (blue), noise genes (grey), and noise metabolites (white) out of top ranked 100 prioritized combined features. Figure 2a suggests that decreasing the relative contribution of the g–g network to the g–m network by setting $\lambda_{g}$¡1, MultiNEP prioritizes more true signal genes and metabolites than that of competing methods under all simulation settings. Figure 2b suggests that increasing the relative contribution of the m–m network to the g–m network by setting $\lambda_{m}$ > 1, MultiNEP prioritizes more true signal metabolites but does not affect much on prioritizing true signal genes. Figure 2 also suggests that optimal values for $\lambda_{g}$ and $\lambda_{m}$ are $\lambda_{g}$ = 0.2, 0.1, 0.05 and $\lambda_{m}$ = 5, 10, 20. We considered different values of $\lambda_{g}$ and $\lambda_{m}$, and results suggest that $\lambda_{g}$ = 0.05 and $\lambda_{m}$ = 20 generate the best prioritization results (Fig. 2c).

To provide a guideline on choices of optimal values of $\lambda_{g}$ and $\lambda_{m}$ in real data analysis, we conducted simulation studies with different degrees of network imbalance, i.e. multiple networks with different numbers of genes and metabolites (see Supplementary Materials and Supplementary Fig. S4). Specifically, we considered simulation settings with networks with fixed 5000 genes and varying number of metabolites to be 30, 60, and 120, being extracted from STRING & STITCH $S^{0}$. Simulation results suggest that the optimal values are $\left(\lambda_{g}=0.01,\lambda_{m}=100\right)$, $\left(\lambda_{g}=0.05,\lambda_{m}=20\right)$, and $\left(\lambda_{g}=0.1,\lambda_{m}=10\right)$, respectively for the three settings. When extracting these networks from PathwayCommons $S^{0}$, the optimal values are $\left(\lambda_{g}=0.005,\lambda_{m}=200\right)$, $\left(\lambda_{g}=0.05,\lambda_{m}=20\right)$, and $\left(\lambda_{g}=0.5,\lambda_{m}=20\right)$, respectively. Overall, smaller values of $\lambda_{g}$ and larger values of $\lambda_{m}$ should be used when $S^{0}$ and *E* are more imbalanced.

#### 3.2.3 Evaluate MultiNEP and competing methods

**Figure 3.**
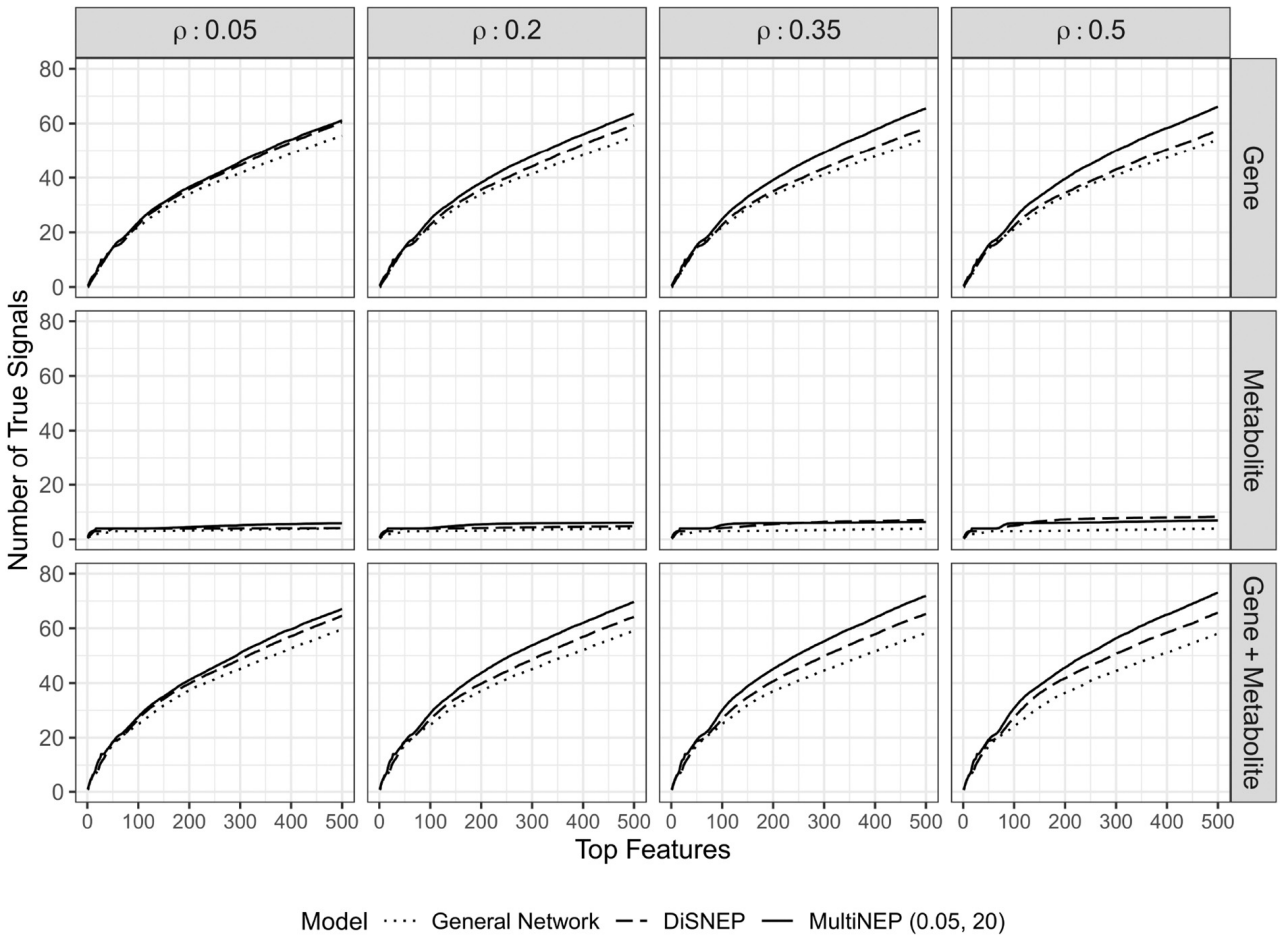
Simulation results of average numbers of identified true signal genes, signal metabolites, and both out of top ranked 1–500 candidate combined features across 100 simulations when signal g–m correlations are ρ=0.05,0.2,0.35,0.5, and $\lambda_{g}=0.05$, $\lambda_{m}=20$ for MultiNEP.

We then fixed $\lambda_{g}=0.05,\lambda_{m}=20$ and evaluated model performance by setting different signal g–m correlations. We observed that MultiNEP (i) consistently prioritizes more combined true signals as well as more true signal genes than that of competing methods across all simulation settings considered; and (ii) slightly prioritizes more true signal metabolites in most of the simulation settings (Fig. 3). When signal g–m correlations are very high with ρ = 0.5, which is higher than that of signal g–g correlations and signal m–m metabolites, MultiNEP prioritizes fewer signal metabolites than that of DiSNEP. This indicates that (i) more efficiently using g–m interactions can help better prioritize disease-associated genes and metabolites; and (ii) when signal g–m correlations (ρ= 0.5) are larger than of signal m–m (ρ= 0.3), increasing relative contributions of m–m to g–m interactions results in not effectively using the stronger g–m interactions, leading to fewer prioritized signal metabolites. Note that in real data, correlations between metabolites are usually higher than that between gene expressions and metabolites.

Table 1 summarizes average IRS scores of identified true signal genes and metabolites by both MultiNEP and DiSNEP, or uniquely by DiSNEP, or MultiNEP out of top ranked 100 features in networks used by General Net: $S_{g}^{0}$, $S_{m}^{0}$, and $S_{gm}^{0}$; by DiSNEP: $S_{Eg}^{\left(1,1\right)}$, $S_{Em}^{\left(1,1\right)}$, and $S_{\mathrm{Egm}}^{\left(1,1\right)}$; and by MultiNEP: $S_{Eg}^{\left(0.05,20\right)}$, $S_{Em}^{\left(0.05,20\right)}$, and $S_{\mathrm{Egm}}^{\left(0.05,20\right)}$, where we denote networks used by DiSNEP or MultiNEP as $S_{E}^{\left(\lambda_{g},\lambda_{m}\right)}$. Here signal g–m correlations were set at ρ=0.35. We notice that (i) true signal genes identified by both DiSNEP and MultiNEP already have high IRS in both $S_{g}^{0}$ and $S_{gm}^{0}$, and their IRS scores are further increased in networks used by DiSNEP and MultiNEP; (ii) for true signal genes uniquely identified by MultiNEP, they have average IRS of 2.76 in $S_{g}^{0}$ but very high IRS of 9.59 in $S_{gm}^{0}$, indicating these genes have much stronger interactions with metabolites, almost 10-fold higher than average; and (iii) for true signal genes uniquely identified by DiSNEP, they have high IRS of 3.44 in $S_{g}^{0}$ but IRS of 1.10 similar as average in $S_{gm}^{0}$. DiSNEP uses these high g–g interactions, while MultiNEP decreases their relative contributions to g–m interactions. When g–m interactions are weak, MultiNEP cannot prioritize them high enough. Similarly, true signal metabolites identified by both DiSNEP and MultiNEP have high IRS in both $S_{m}^{0}$ and $S_{gm}^{0}$. True signal metabolites uniquely identified by MultiNEP have strong m–m interactions with IRS = 1.79 in $S_{m}^{0}$ but weak g–m interactions with IRS = 0.84 in $S_{gm}^{0}$. After reweighting, these IRS increase. These results demonstrate that MultiNEP could prioritize signal metabolites by using more m–m interactions buried by overwhelming g–m interactions in the imbalanced multi-omics network which are missed by DiSNEP.

**Table 1.** Simulation results of IRS of identified true signal genes out of top ranked 100 features across 100 simulations with signal g–m correlations ρ = 0.35.

		Average IRS of identified true signal G (average no. of true signal G)				Average IRS of identified true signal M (average no. of true signal M)		
		DiSNEP&MultiNEP (20.45)	DiSNEP only (2.06)	MultiNEP only (4.14)		DiSNEP&MultiNEP (4.15)	DiSNEP only (0)	MultiNEP only (1.26)
General Net	$S_{g}^{0}$	5.38	3.44	2.76	$S_{m}^{0}$	2.85		1.79
	$S_{gm}^{0}$	4.25	1.10	9.59	$S_{gm}^{0}$	5.63		0.84
DiSNEP	$S_{Eg}^{\left(1,1\right)}$	12.95	4.96	3.19	$S_{Em}^{\left(1,1\right)}$	2.40		1.92
	$S_{\mathrm{Egm}}^{\left(1,1\right)}$	3.25	2.28	2.77	$S_{\mathrm{Egm}}^{\left(1,1\right)}$	5.95		1.03
MultiNEP	$S_{Eg}^{\left(0.05,20\right)}$	12.68	3.97	7.97	$S_{Em}^{\left(0.05,20\right)}$	2.48		1.94
	$S_{\mathrm{Egm}}^{\left(0.05,20\right)}$	3.16	2.40	3.44	$S_{\mathrm{Egm}}^{\left(0.05,20\right)}$	6.48		1.58

### 3.3 Real data applications

We applied MultiNEP and competing methods to prioritize candidate disease-associated gene expressions and metabolites for two human cancers, the Dana-Farber Cancer Institute/Harvard Cancer Center (DF/HCC) Prostate Cancer Cohort (Oh *et al.* 2006, Xu *et al.* 2022), and the GEO Breast Cancer cohort (GSE37751) (Terunuma *et al.* 2014).

#### 3.3.1 DF/HCC prostate cancer cohort

##### 3.3.1.1 A multi-omics general network $S_{0}$

We obtained a general multi-omics network from STRING and STITCH databases as described in simulation studies and kept genes and metabolites that are also in prostate cancer disease omics profiles. There are 18 009 genes and 203 metabolites with 5 398 067 g–g interactions, 4715 m–m interactions and 82 529 g–m interactions in $S_{0}$ (Supplementary Table S2).

##### 3.3.1.2 Disease multi-omics profiles

Available information in the DF/HCC Prostate Cancer Cohort are described in Oh *et al.* (2006). We used 94 tumor and 48 normal-adjacent tissue samples (with 48 with tumor and normal-adjacent pairs) with gene expression and metabolite abundance data as described in (Xu *et al.* 2022). We constructed the disease similarity matrix *E* with correlations among 18 009 gene expressions and 203 metabolites using omics profiles of 94 tumors and 48 normal-adjacent samples, and calculated disease association scores *v* using paired t-test with 48 matched tumor and normal-adjacent pairs. See Supplementary Materials for detailed data processing pipeline.

##### 3.3.1.3 Signal prioritization

Suggested by simulation studies, we set $\lambda_{g}$=0.05 and $\lambda_{m}$=20, and considered 632 prostate cancer-related genes (DOID: 10283) based on DisGeNET as gold standards. No reliable list of prostate cancer-related metabolites is available. We thus only evaluated model performance using numbers of prostate cancer-related genes prioritized by MultiNEP and competing methods within top ranked 1 to 500 genes.

**Figure 4.**
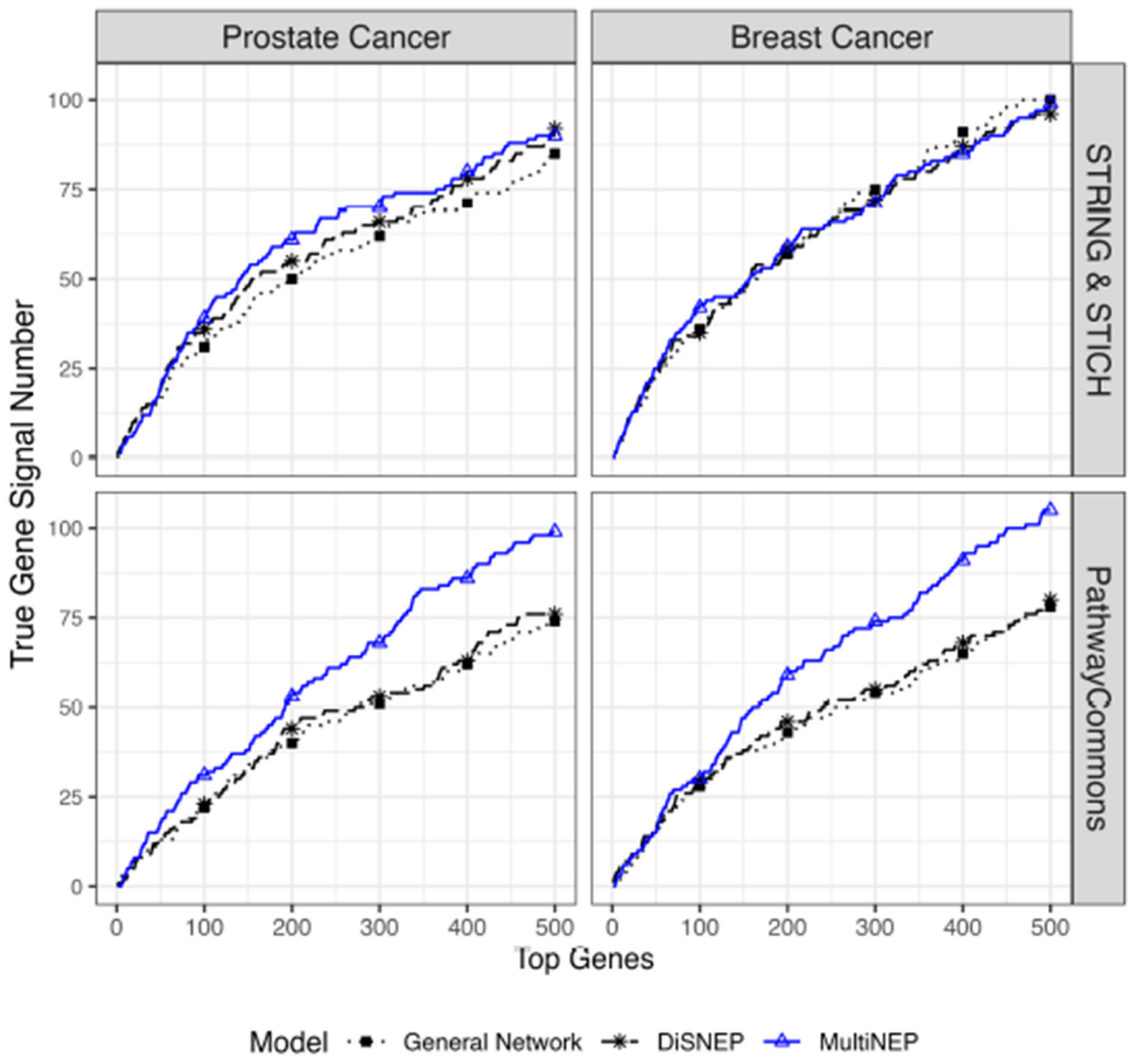
Real data application results with numbers of identified prostate cancer-related genes (left panel) and breast cancer-related genes (right panel) based on DisGeNet within top ranked 1–500 candidate genes. We set $\lambda_{g}$ = 0.05 and $\lambda_{m}$ = 20 for MultiNEP with STRING & STITCH network (prostate cancer: 18 009 genes and 203 metabolites; breast cancer: 17 202 genes and 224 metabolites), and $\lambda_{g}$ = 0.005 and $\lambda_{m}$ = 200 for MultiNEP with PathwayCommons network (prostate cancer: 18 490 genes and 111 metabolites; breast cancer: 17 609 genes and 108 metabolites).

As shown in Figure 4 (left panel), MultiNEP consistently prioritized more prostate cancer-related genes based on the DisGeNET database out of top ranked 1 to 500 candidate genes than that of the competing methods. We further investigated results from top ranked 200 candidate genes. MultiNEP and DiSNEP prioritized 61 and 55 prostate cancer-related genes, respectively. Of those, 49 genes overlap, 12 were uniquely identified by MultiNEP and 6 by DiSNEP. Out of the 49 overlapping genes, many are important cancer genes or prostate cancer genes, such as TP53 (Jillson *et al.* 2021), CTNNB1 (Patel *et al.* 2020), EGFR (Nastały *et al.* 2020), etc. We calculated IRS for the 49 overlapping genes, 12 uniquely identified by MultiNEP and 6 by DiSNEP (Supplementary Table S4). The 49 overlapping genes have high IRS in general networks ($S_{g}^{0}$, $S_{gm}^{0}$) and can be identified by both methods using original g–g or g–m interactions. These genes have even higher IRS in enhanced ($S_{Eg}^{\left(1,1\right)}$, $S_{\mathrm{Egm}}^{\left(1,1\right)}$) and reweighted networks ($S_{Eg}^{\left(0.05,20\right)}$, $S_{\mathrm{Egm}}^{\left(0.05,20\right)}$). The 6 genes uniquely identified by DiSNEP have high IRS of 3.53 in $S_{g}^{0}$ but relatively low IRS of 1.85 in $S_{gm}^{0}$. DiSNEP uses strong g–g interactions to prioritize them to the top. Instead, MultiNEP lowered the relative contribution of g–g to g–m interactions and cannot identify these genes when g–m interactions are relatively weak. The 12 genes uniquely identified by MultiNEP have high IRS of 3.15 in $S_{g}^{0}$ and high IRS of 7.34 in $S_{gm}^{0}$. MultiNEP puts even more weights on the already strong g–m interactions, resulting in higher ranks of these genes. One gene uniquely identified by MultiNEP, the NOS3 gene, is a known prostate cancer-related gene and is associated with tumor angiogenesis, proliferation, and invasiveness (Zou *et al.* 2021). NOS3 ranks 55th by MultiNEP, 419th by DiSNEP and 687th by General Network. NOS3 interacts with 54 metabolites and 1796 genes in $S^{0}$, with 85 metabolites and 4 379 genes in $S_{E}^{\left(1,1\right)}$, and with 111 metabolites and 12 419 genes in $S_{E}^{\left(0.05,20\right)}$.

##### 3.3.1.4 Sensitivity analysis using a different general network $S_{0}$

We investigated how different general network $S_{0}$ affects prioritization results. We used $S^{0}$ from PathwayCommons (Cerami *et al.* 2011) where all interactions are binary. There are 18 490 genes and 111 metabolites with 1 926 474 g–g interactions, 462 m–m interactions and 7023 g–m interactions in PathwayCommons $S^{0}$ (Supplementary Table S2). We set $\lambda_{g}=0.005$ and $\lambda_{m}=200$ for MultiNEP when using PathwayCommons $S^{0}$ based on simulation results in Supplementary Figure S4. We observed similar results as that using the STRING & STITCH $S^{0}$, but bigger improvements (bottom panel in Fig. 4). This is because many prostate cancer-related signal genes have stronger g–m interactions in PathwayCommons $S^{0}$ than in STRING & STITCH $S^{0}$ indicated by IRS (for detailed results, see Supplementary Materials and Supplementary Table S4).

#### 3.3.2 GSE37751 breast cancer cohort

We also applied MultiNEP and competing methods to the GEO Breast Cancer cohort (GSE37751) (Terunuma *et al.* 2014) with gene expression and metabolite data and observed similar results as for the prostate cancer cohort (Supplementary Table S4 and Fig. 4). Detailed results are in the Supplementary Materials.

## 4 Discussion

We developed MultiNEP, a network-assisted method to simultaneously prioritize candidate disease-associated gene expressions and metabolites using a multi-omics network. MultiNEP accounts for the imbalances in numbers of gene expressions and metabolites in networks to more effectively use g–m interactions that are not used in existing network-assisted methods that prioritize candidate disease-associated gene expressions and metabolites separately. Gene–metabolite crosstalks were observed in many human diseases, especially in cancers (Yoshida 2015). Using additional g–m interactions in network-assisted methods could improve prioritization of candidate disease-associated gene expressions and metabolites. Because of the much larger number of gene expressions than that of metabolites, direct applications of existing network-assisted methods with a multi-omics network, such as DiSNEP (Ruan and Wang 2021), do not achieve desired improvements in prioritization (Supplementary Fig. S1). The proposed MultiNEP framework employs two weighting parameters to down-weight the relative contribution of the g–g network to the g–m network ($0<\lambda_{g}$¡1) and up-weight the relative contribution of the m–m network to the g–m network ($\lambda_{m}$¿1) to account for network imbalance so that both within- and between-omics interactions are efficiently used.

In simulation studies, we observed that MultiNEP prioritizes more true signal genes (Fig. 2a) than competing methods through utilizing g–m interactions that are outnumbered by g–g interactions if we down-weight the relative contribution of the g–g to the g–m network by setting the proposed parameter $0<\lambda_{g}<1$. Similarly, MultiNEP slightly prioritizes more true signal metabolites (Fig. 2b) when we up-weight the relative contribution of the m–m to the g–m network by setting the proposed parameter $\lambda_{m}>1$. The improvement is much smaller due to the small number of metabolites in a multi-omics network (Fig. 3). We also noticed in simulation studies that when signal g–m correlations are larger (such as ρ=0.5) than signal m–m correlations (such as ρ=0.3), MultiNEP prioritizes fewer true metabolites than that of DiSNEP. This is expected because increasing relative contributions of m–m to g–m interactions when g–m interactions are stronger will lead to fewer prioritized true signal metabolites. In real data, g–m correlations are usually smaller than g–g and m–m correlations, our current weighting scheme is expected to help prioritization. Real data applications to two cancer cohorts also suggested improved performance of MultiNEP than competing methods that ignore network imbalance (Fig. 4). In sensitivity analyses using a different general network from PathwayCommons, we confirmed the superior performance of MultiNEP. Results using the metric IRS (interaction ratio score) also suggest that those prioritized candidate disease-associated genes uniquely identified by MultiNEP interact with more metabolites where only MultiNEP can efficiently use these strong g–m interactions and prioritize them to higher ranks (Supplementary Table S4). Unfortunately, due to the limitation in current disease-associated metabolite databases, we cannot evaluate the performance of MultiNEP in prioritizing candidate disease-associated metabolites in real data analyses.

We used the same weighting scheme for $S^{0}$ and *E* because the two matrices have the same imbalance issue with the same number of genes and number of metabolites. We also investigated if reweighting both $S^{0}$ and *E* is better than reweighting only $S^{0}$, denoted as MultiNEP $S^{0}$ by conducting additional simulation studies (results are in Supplementary Fig. S2). We observed similar improvements of MultiNEP $S^{0}$ over competing methods, while MultiNEP consistently prioritizes more signal genes and metabolites than MultiNEP $S^{0}$. Moreover, when signal g–m correlations in disease omics profiles increase, MultiNEP has bigger improvements than MultiNEP $S^{0}$, indicating that the imbalance issue exists in both $S^{0}$ and *E*.

Although we focused on prioritizing candidate disease-associated gene expressions and metabolites in this study, the MultiNEP framework is general. It can be used to prioritize metabolites together with other omics types where network imbalance issues exist, such as combining metabolomics with copy number alterations or DNA methylations. The developed MultiNEP framework is implemented in an R package, which is freely available at https://github.com/Karenxzr/MultiNEP.

## Supplementary Material

btad333_Supplementary_Data

## Data Availability

DF/HCC prostate cancer cohort data used in this article is available at (https://github.com/Karenxzr/MultiModalPC) from Xu et al. (2022). The breast cancer cohort data used in this article can be downloaded from (https://www.ncbi.nlm.nih.gov/geo/) (accesion number: GSE37751) and supplementary data from Terunuma et al. (2014).
