# Supplementary material for: MultiNEP: a multi-omics network enhancement framework for prioritizing disease genes and metabolites simultaneously
Source: Bioinformatics. 2023 May 22;39(6):btad333. doi: 10.1093/bioinformatics/btad333 (PMC10250081; doi:10.1093/bioinformatics/btad333)
Supplement: btad333_Supplementary_Data [file btad333_supplementary_data.pdf]

S1 Supplementary materials

S1.1 Choice of λ_g and λ_m for disease profiles with different feature numbers

We performed additional simulation studies to guide selecting values of λ_g and λ_m in real data analysis, where different ratios of gene numbers to metabolite numbers will result in different degrees of network imbalance. We set gene numbers ($\#g$) as 5,000 and metabolite numbers ($\#m$) as 30, 60, and 120, respectively, and considered both STRING & STITCH S^0 and PathwayCommons S^0. The selection of $\#g = 5,000$ and $\#m = 30$ is to mimic the imbalance of DF/HCC cohort data when using PathwayCommons S^0. The selection of $\#g = 5,000$ and $\#m = 60$ is to mimic the imbalance of DF/HCC cohort data when using STRING & STITCH S^0. The methods of obtaining the general gene network are the same with those described in the main manuscript with the only exception that S^0 might come from PathwayCommons. When using STRING & STITCH S^0, we randomly selected 30, 60, and 120 metabolites from the 203 metabolites in the STITCH network, with 6, 12, and 24 were randomly set as true signal metabolites and the remaining as noises. When using PathwayCommons S^0, we randomly selected 30 and 60 metabolites from the 111 metabolites in the PathwayCommons network, with 6 and 12 were randomly set as true signal metabolites and the remaining as noises. To make the simulation setting of $\#m = 120$ consistent for STRING & STITCH S^0 and PathwayCommons S^0, we randomly selected 24 metabolites from PathwayCommons S^0 and set as true signal metabolites. 96 metabolites were randomly sampled with replacements from the remaining 87 metabolites as noises. Correlations between signal genes and signal metabolites were set as $\rho = 0.5$. All other settings are the same with those in the main manuscript. We compared average numbers of true signal genes and true signal metabolites identified by MultiNEP using different weighting parameters within top 100 prioritized overall features. A series of λ_g and λ_m values were considered: ($\lambda_g = 0.2$, $\lambda_m = 5$), ($\lambda_g = 0.1$, $\lambda_m = 10$), ($\lambda_g = 0.05$, $\lambda_m = 20$), ($\lambda_g = 0.02$, $\lambda_m = 50$), ($\lambda_g = 0.01$, $\lambda_m = 100$), ($\lambda_g = 0.05$, $\lambda_m = 200$), and ($\lambda_g = 0.002$, $\lambda_m = 500$).

S1.2 Choice of denoising thresholds for S_E sub-networks

In step2 of the MultiNEP framework, the enhanced disease-specific network $S^{t,sym}$ needs to be further denoised. Because there are 3.3% and 3.5% edges in prostate and breast cancer S^0_g; 23.0% and 17.7% edges in prostate and breast cancer S^0_m; and 2.3% and 1.6% edges in prostate and breast cancer S^0_{gm}, we chose 5% and 30% as denoising thresholds for $S^{t,sym}_g$ and $S^{t,sym}_m$, respectively. Since g-m interactions are much less studied, we kept more g-m interactions (15%) in $S^{t,sym}_{gm}$ (**Tables S2, S3**).

S1.3 Data Processing

DF/HCC Prostate Cancer Cohort For metabolites, we followed the preprocessing pipeline as in Penney et al. [2021] implemented in the R package maplet Chetnik et al. [2021]. Briefly, we started with 222 metabolites detected from three different batches of experiments. We excluded 8 metabolites with more than 50% of missing, leaving us with 214 metabolites. After removing batch effects by correcting data to the run day median and performing quotient normalizations Dieterle et al. [2006], we imputed missing metabolites using K-nearest neighbor method Do et al. [2018]. We also excluded 11 metabolites that are not in the general network S_0 and ended up with 203 metabolites for analysis. For gene expressions, we used the robust multichip average (RMA) algorithm Irizarry [2003] from oligo R package Carvalho and Irizarry [2010] to normalize gene expressions and transformed to $log2$ scale, and only kept the probe with the largest variance for those with the same gene symbol annotation. We removed batch effects using 'removeBatchEffect' function from the R package limma Ritchie et al. [2015]. We ended with 29,054 genes where 18,009 of them are in the general network S_0. Note that, MultiNEP does not require gene expression and metabolite data to be normalized.

GSE37751 Breast Cancer Cohort There are 350 identified and pre-processed metabolites, among which 224 can be mapped in the general metabolite network. We downloaded the processed gene expression data, and transformed into $log2$ scale. We used the probes with maximum expression variance across samples to represent gene expressions if the gene has multiple probes. There are 23,199 genes with expression profiles, among which 17,202 genes are also in the general network.

S1.4 Application using GSE37751 Breast Cancer Cohort

A Multi-omics general network A general multi-omics network S^0 was obtained from STRING and STITCH databases. We kept only genes and metabolites that are also in the breast cancer disease omics profiles. Thus, the general network S^0 has 17,202 genes, 224 metabolites, with 10,368,398 g-g interactions, 8,862 m-m interactions and 61,589 g-m interactions (**Table S3**).

Disease multi-omics profiles The omics profiles of GSE37751 Breast Cancer Cohort include DNA methylation, gene expression, and metabolome of fresh-frozen human breast tumors [Terunuma et al., 2013]. We only included 60 tumor and 47 normal-adjacent breast tissue samples (with 45 matched tumor and normal-adjacent pairs) with both gene expressions and metabolite abundances. Disease-specific similarity matrix E was constructed using 17,202 gene expressions and 224 metabolites of all 107 samples (60 tumor samples and 47 normal-adjacent tissue samples). Disease association scores v were generated using paired t statistics of 45 matched tumor and normal-adjacent pairs.

Signal prioritization We set $\lambda_g = 0.05$, and $\lambda_m = 20$ when applying MultiNEP to prioritize candidate breast cancer-related genes and metabolites. The 490 breast cancer (C0678222) related genes from DisGeNET were used as gold standards. We evaluated model performance by comparing the numbers of breast cancer-related genes prioritized by MultiNEP and competing methods within top ranked 1 to 500 candidate genes.

As shown in the top right panel of **Figure 4**, the general network, DiSNEP and MultiNEP prioritized similar numbers of breast cancer-related genes within top ranked 1 to 500 genes. We investigated reasons for the similar performances in section S1.5. Similar as in prostate cancer cohort, we investigated top ranked 200 genes identified by MultiNEP and DiSNEP. MultiNEP and DiSNEP prioritized 59 and 58 breast cancer-related genes. Of those 54 genes overlapping, 5 were uniquely identified by MultiNEP, and DiSNEP identified 4. We similarly calculated IRS for the 54 overlapping genes, 5 genes uniquely identified by MultiNEP and 4 genes uniquely identified by DiSNEP as in the application using the DF/HCC prostate cancer cohort, and observed similar patterns of results. Specifically, the 54 overlapping genes already have high IRS in S^0_g and S^0_{gm}, and can be identified by both DiSNEP and MultiNEP using either g-g interactions or g-m interactions. These genes have even higher IRS in $S^{(1,1)}_{Eg}$, $S^{(0.05,20)}_{Eg}$, $S^{(1,1)}_{Egm}$, and $S^{(0.05,20)}_{Egm}$ used by MultiNEP and DiSNEP. The 4 genes uniquely identified by DiSNEP have high IRS of 4.00 in S^0_g but low IRS of 0.62 in S^0_{gm}. Without adjusting for relative contributions of g-g interactions to g-m interactions, DiSNEP can use their strong g-g interactions and overlook their weak g-m interactions to prioritize them with high ranks. Instead, MultiNEP lowered the relative contribution of their strong g-g interactions to their weak g-m interactions, and thus cannot identify them. The 5 genes uniquely identified by MultiNEP have high IRS of 3.27 in S^0_g and higher IRS of 9.36 in S^0_{gm}, so addressing more on their stronger g-m interactions relative to their g-g interactions can result in higher ranks. Above results again confirmed the ability of MultiNEP to handle network imbalance and boost signal prioritization performance.

Sensitivity analysis using a different general network S^0 For sensitivity analysis, we obtained the general multi-omics network from PathwayCommons [Cerami et al., 2010], which has 17,609 genes, 108 metabolites, 924,601 g-g interactions, 174 m-m interactions, and 5,216 g-m interactions (**Table S3**). We set $\lambda_g = 0.005$ and $\lambda_m = 200$ for MultiNEP when using PathwayCommons S^0. Similarly, MultiNEP consistently outperforms competing methods and identified more breast cancer-related genes.

S1.5 MultiNEP performance improvements with different S^0

In both prostate and breast cancer cohorts, we observed bigger improvements of MultiNEP over competing methods when using PathwayCommons S^0 than STRING&STITCH S^0. For simplicity, we use General Net (PC), DiSNEP (PC), MultiNEP (PC) to denote methods when using PathwayCommons S^0, and use General Net (SS), DiSNEP (SS), and MultiNEP (SS) to denote methods when using STRING&STITCH S^0. We investigated top ranked 200 prioritized candidate prostate cancer-related genes in details. Out of top 200 prioritized candidate prostate cancer-related genes, DiSNEP(PC) and MultiNEP(PC) identified 44 and 53 prostate cancer-associated genes based on DisGeNET (MultiNEP has 20% more.), while DiSNEP(SS) and MultiNEP (SS) identified 55 and 61 prostate cancer-associated genes based on DisGeNET (MultiNEP has 10% more.). Similarly among top ranked 200 prioritized candidate breast cancer-related genes, DiSNEP(PC)

and MultiNEP(PC) identified 46 and 59 breast cancer-associated genes based on DisGeNET (MultiNEP has 25% more.), while DiSNEP(SS) and MultiNEP (SS) identified 58 and 59 breast cancer-associated genes based on DisGeNET (MultiNEP has 3% more.).

We investigated why there is a bigger improvement when using PathwayCommons S^0. To do so, we examined the 16 prostate cancer-related genes and 20 breast cancer-related genes identified only by MultiNEP (PC), but not by the other five methods (General Net (PC), DiSNEP (PC), General Net (SS), DiSNEP (SS), and MultiNEP (SS)) among top 200 prioritized candidate genes based on DisGeNET. Using the 16 prostate cancer-related gene as an example, as shown in **Table S5**, these 16 genes have weak IRS in STRING & STITCH S^0_g (IRS=2.22) and S^0_{gm} (IRS=3.07). The g-g and g-m interactions are not strong enough to help prioritize these genes using either DiSNEP (SS) or MultiNEP (SS). On the contrary, these 16 genes have stronger IRS in PathwayCommons S^0_g (IRS=3.88) and extremely strong IRS in S^0_{gm} (IRS=24.52). These strong g-m interactions can only be efficiently used by MultiNEP (PC) after giving more weights on g-m interactions. Similar patterns can be observed in the 20 breast cancer-related gene uniquely identified by MultiNEP (PC).

S1.6 MultiNEP performance improvements with different omics profiles

As observed in **Figure 4**, with PathwayCommons, MultiNEP(SS) outperforms DiSNEP(SS) and General Net(SS) using either prostate cancer or breast cancer data. With STRING&STITCH, all three have similar performance especially when using breast cancer data (**Figure 4**). We thus investigated rankings of IRS scores of genes that are also in STRING&STITCH or PathwayCommons, which we separated these genes into prostate/breast cancer-related genes based on the DisGeNET database and other noise genes. We considered rankings of these genes in terms of IRS. Recall that IRS are interaction ratio scores that measure if individual features (genes/metabolites) have stronger/weaker interactions relative to the average. That is, we investigated (1) differences in rankings of cancer-related genes in GeneralNET S^0 and that in DiSNEP $S^{(1,1)}_{Eg}$, which could reflect how much information disease omics profiles help in enhancing the general network, and could be used to understand differences in performance between GeneralNET and DiSNEP, and (2) differences in rankings of cancer-related genes in DiSNEP $S^{(1,1)}_{Eg}$ and that in STRING&STITCH MultiNEP $S^{(0.05,20)}_{Eg}$ (or that in PathwayCommons MultiNEP $S^{(0.005,200)}_{Eg}$), which could reflect how much information the reweighting steps bring, and could be used to explain differences in performance between DiSNEP and MultiNEP.

For item (1), the mean differences in rankings of cancer-related genes GeneralNet - DiSNEP are:

- for STRING&STITCH and prostate cancer: 3.84 (±1215), this suggests that these cancer-related genes rank higher in terms of IRS in DiSNEP after enhancement using omics data;
- for STRING&STITCH and breast cancer: -93.4 (±818), this suggests that these cancer-related genes rank lower in term of IRS in DiSNEP after enhancement using omics data;
- for PathwayCommons and prostate cancer: 37.5 (±1251);
- for PathwayCommons and breast cancer: -12.1(±988).

These results explain the bigger improvements of DiSNEP over that of GeneralNET in prostate cancer data than that in breast cancer data. When comparing STRING&STITCH and PathwayCommons for prostate cancer data, the change in ranking is bigger on average for PathwayCommons (mean=37.5) than that for STRING&STITCH (mean=3.84), which explains the bigger improvements using PathwayCommons.

For item (2), the mean differences in rankings of cancer-related genes DiSNEP - MultiNEP are:

- for STRING&STITCH and prostate cancer: 36.8 (±914);
- for STRING&STITCH and breast cancer: 1.43 (±693);
- for PathwayCommons and prostate cancer: 182 (±1505);
- for PathwayCommons and breast cancer: 124 (±1587).

These results explain the bigger improvements of MultiNEP over that of DiSNEP in prostate cancer data than that in breast cancer data. When comparing STRING&STITCH and PathwayCommons for prostate cancer data, the change in ranking is much bigger on average for PathwayCommons (mean=182) than that for STRING&STITCH (mean=36.8)

Overall, these results explain bigger improvements using PathwayCommons than using STRING&STITCH in general, and bigger improvements for prostate cancer data than that for breast cancer data in general. That also explains that the performance of three methods is similar in Figure 4 top right, that is, for breast cancer data using STRING&STITCH.

S1.7 Statistics of computational times

We use the DF/HCC prostate cancer data as an illustrative example to describe the computational times of MultiNEP and competing methods. S^0 from STRING and STITCH databases with 18,009 genes and 203 metabolites was used. The general network finished computing in 1.05 mins without involving the step of network enhancement. DiSNEP finished network enhancement within 13 iterations and took 57.39 mins. MultiNEP finished network enhancement within 16 iterations and took 70.68 mins. MultiNEP and DiSNEP used 4 CPU cores for parallel computing.

S2 Supplementary tables and figures

Table S1: Descriptive statistics of two general networks.

**Network Resource**	**Network**	**# Nodes**	**# Edges**	**Edge%***	**Edge Weights†**
String & Stitch	Gene-Gene	19,385	5,969,249	3.18%	216 (175, 309)
	Metabolite-Metabolite	389,393	8,852,909	0.01%	230(175, 353)
	Gene-Metabolite	408,778	15,473,939	0.20%	221(178, 288)
PathwayCommons	Gene-Gene	19,572	1,040,197	0.54%	
	Metabolite-Metabolite	11,346	14,750	0.02%	
	Gene-Metabolite	30,918	654,005	0.29%	

*: percentage of edges with non-zero weights out of all possible edges of a network with # nodes
†: edges with non-zero weights: median (25th percentile, 75th percentile).

Table S2: Descriptive statistics of trimmed two general networks for the DF/HCC prostate cancer cohort.

**Network Resource**	**Network**	**# Nodes**	**# Edges**	**Edge%***	**Edge Weights†**
String & Stitch	Gene-Gene	18,009	5,398,067	3.33%	216 (175, 309)
	Metabolite-Metabolite	203	4,715	23.0%	388 (218, 900)
	Gene-Metabolite	18,212	82,529	2.26%	256(178, 644)
PathwayCommons	Gene-Gene	18,490	963,237	0.56%	
	Metabolite-Metabolite	111	231	3.78%	
	Gene-Metabolite	19,051	7,023	0.34%	

*: percentage of edges with non-zero weights out of all possible edges of a network with # nodes
†: edges with non-zero weights: median (25th percentile, 75th percentile).

Table S3: Descriptive statistics of trimmed two general networks for the GSE37751 Breast Cancer cohort.

Network Resource	Network	# Nodes	# Edges	Edge%*	Edge Weights†
String & Stitch	Gene-Gene	17,202	5,184,199	3.50%	217 (175, 310)
	Metabolite-Metabolite	224	4,431	17.74%	329 (209, 718)
	Gene-Metabolite	17,426	61,589	1.60%	229(173, 432)
PathwayCommons	Gene-Gene	17,609	924,601	0.60%	
	Metabolite-Metabolite	108	174	3.01%	
	Gene-Metabolite	17,717	5,216	0.27%	

*: percentage of edges with non-zero weights out of all possible edges of a network with # nodes
†: edges with non-zero weights: median (25th percentile, 75th percentile).

Table S4: Average IRS of identified prostate cancer-related / breast cancer-related genes based on DisGeNet within top ranked 200 genes.

		Average IRS of identified prostate cancer-related G (average # of prostate cancer-related G)			Average IRS of identified breast cancer-related G (average # of breast cancer-related G		
		Both DiSNEP & MultiNEP (49)	DiSNEP Only (6)	MultiNEP Only (12)	Both DiSNEP & MultiNEP (54)	DiSNEP Only (4)	MultiNEP Only (5)
General Net	S_g^0	5.98	4.16	3.36	5.78	4.00	3.27
	S_{gm}^0	4.37	1.78	9.35	6.37	0.62	9.36
DiSNEP	$S_{Eg}^{(1,1)}$	13.03	7.77	5.43	12.76	7.30	4.90
	$S_{Egm}^{(1,1)}$	5.16	3.26	2.73	6.10	5.48	5.02
MultiNEP	$S_{Eg}^{(0.05,20)}$	12.78	6.27	9.25	12.70	5.70	7.49
	$S_{Egm}^{(0.05,20)}$	3.10	2.07	2.94	4.57	2.81	3.78

Table S5: Average IRS of genes uniquely identified by MultiNEP (PC) but not by the other 5 methods out of top ranked 200 prioritized candidate genes. We set $\lambda_g = 0.005, \lambda_m = 200$ for MultiNEP (PC), and $\lambda_g = 0.05, \lambda_m = 20$ for MultiNEP (SS).

	Average IRS of genes (prostate cancer) uniquely identified by MultiNEP using PathwayCommons S^0 (16 genes)				Average IRS of genes (breast cancer) uniquely identified by MultiNEP using PathwayCommons S^0 (20 genes)			
	STRING & STITCH		PathwayCommons		STRING & STITCH		PathwayCommons	
General Net	S_g^0	2.22	S_g^0	3.88	S_g^0	2.20	S_g^0	3.25
	S_{gm}^0	3.07	S_{gm}^0	24.52	S_{gm}^0	4.38	S_{gm}^0	32.07
DiSNEP	$S_{Eg}^{(1,1)}$	2.13	$S_{Eg}^{(1,1)}$	4.55	$S_{Eg}^{(1,1)}$	2.03	$S_{Eg}^{(1,1)}$	2.81
	$S_{Egm}^{(1,1)}$	1.60	$S_{Egm}^{(1,1)}$	4.87	$S_{Egm}^{(1,1)}$	2.52	$S_{Egm}^{(1,1)}$	4.60
MultiNEP	$S_{Eg}^{(0.05,20)}$	2.21	$S_{Eg}^{(0.005,200)}$	18.92	$S_{Eg}^{(0.05,20)}$	2.26	$S_{Eg}^{(0.005,200)}$	18.63
	$S_{Egm}^{(0.05,20)}$	1.51	$S_{Egm}^{(0.005,200)}$	5.47	$S_{Egm}^{(0.05,20)}$	1.79	$S_{Egm}^{(0.005,200)}$	4.06

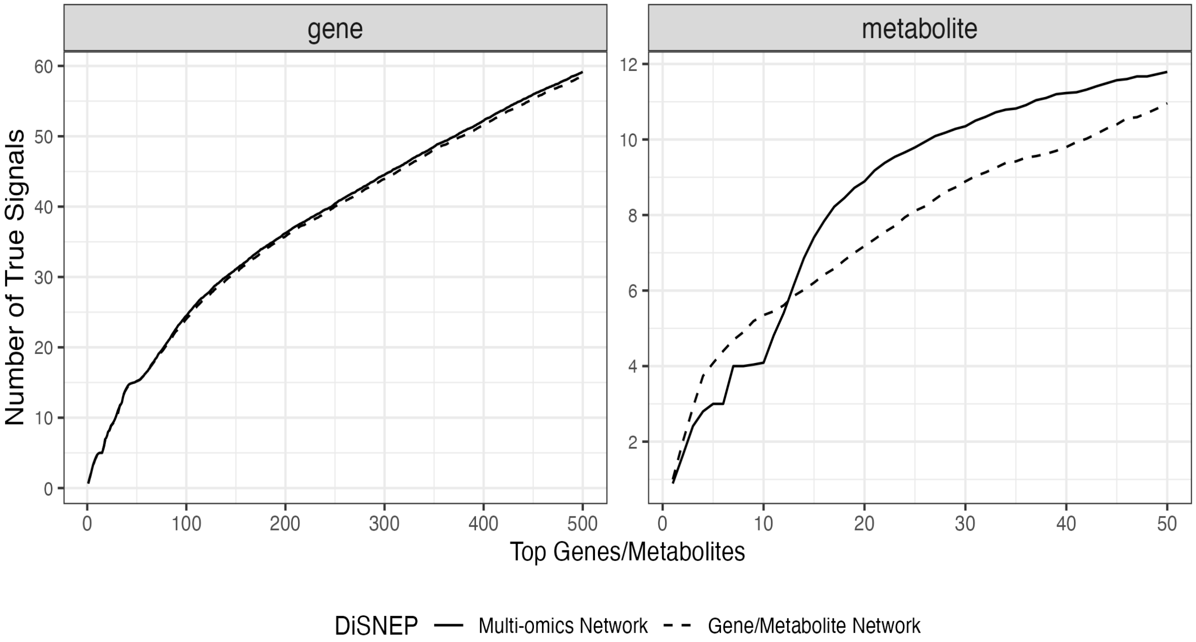

Figure S1: Simulation results comparing extended DiSNEP when using a multi-omics network to the original DiSNEP when using a single-omics network. Dashed lines are average numbers of identified true gene signals out of top ranked 1 to 500 prioritized candidate genes (the left panel), and average numbers of identified true metabolite signals out of top ranked 1 to 50 prioritized candidate metabolites (the right panel) using the original DiSNEP with a single-omics network. Solid lines are that when using the extended DiSNEP with a multi-omics network out of top ranked 1-500 genes, and out of top ranked 1-50 metabolites. All numbers are averaged over 100 simulations, with correlations between signal genes and signal metabolites set at $\rho = 0.35$.

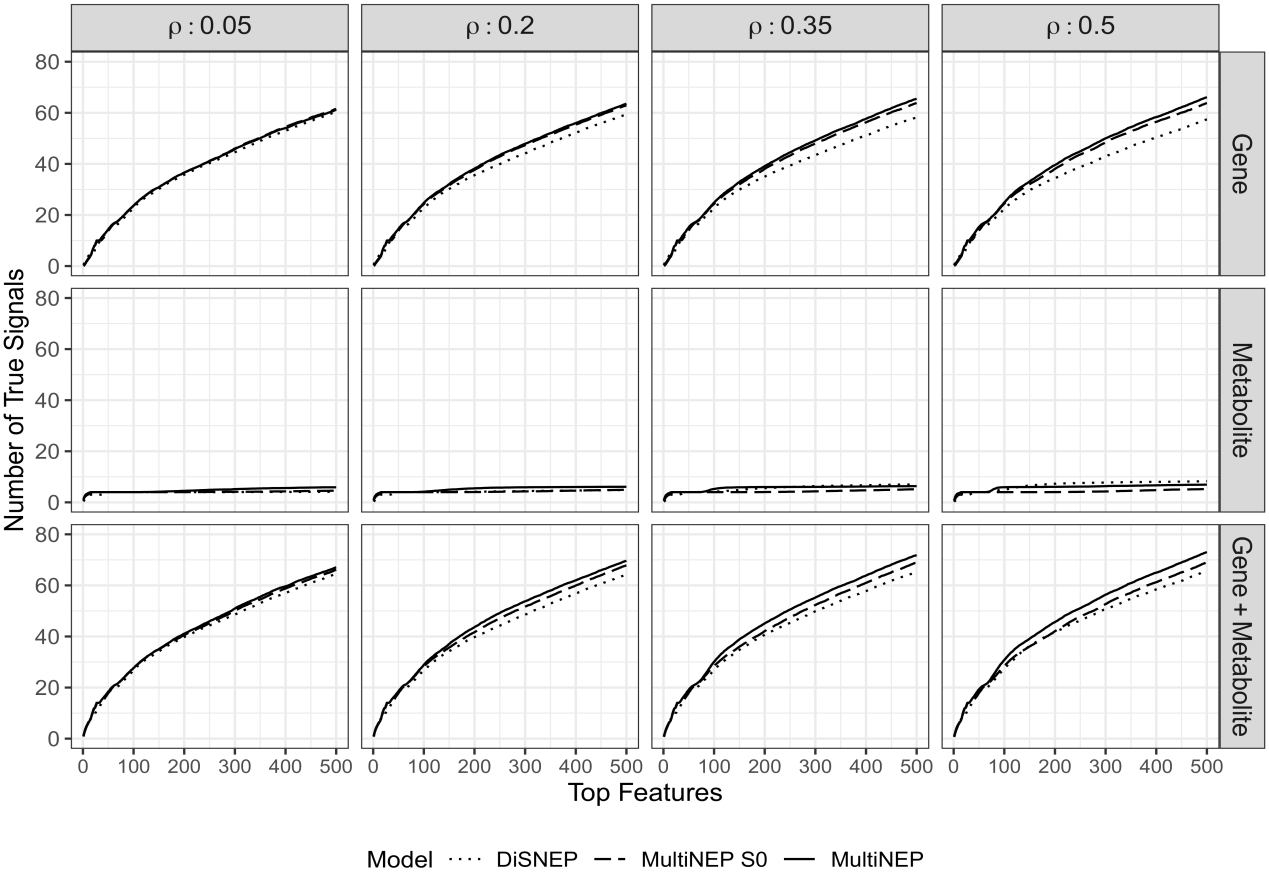

Figure S2: Simulation results comparing performance of MultiNEP S^0 that only reweights S^0 to that of MultiNEP that reweights both S^0 and E. Displayed are average numbers of identified true signal genes, signal metabolites, and both (genes and metabolites) out of top ranked 1 to 500 combined features across 100 simulations when correlations between signal genes and signal metabolites were set at $\rho = 0.05, 0.2, 0.35, 0.5$. We set $\lambda_g = 0.05, \lambda_m = 20$ for both MultiNEP S^0 and MultiNEP.

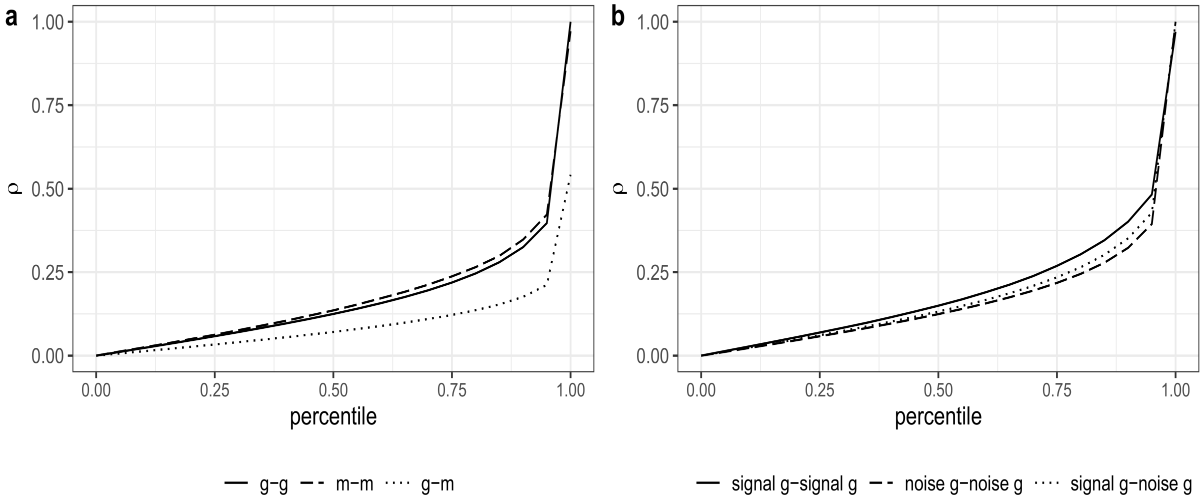

Figure S3: Distribution of pairwise correlations in DF/HCC Prostate Cancer Cohort omics profiles. **a.** Displayed are percentile values of ρ between 18,009 genes (solid line), between 203 metabolites (long dashed line), and between 18,009 genes and 203 metabolites (short dashed line). **b.** Displayed are percentile values of ρ between 632 signal genes based on DisGeNET (solid line), between 17,377 noise genes (long dashed line), and between 632 signal genes and 17,377 noise genes (short dashed line).

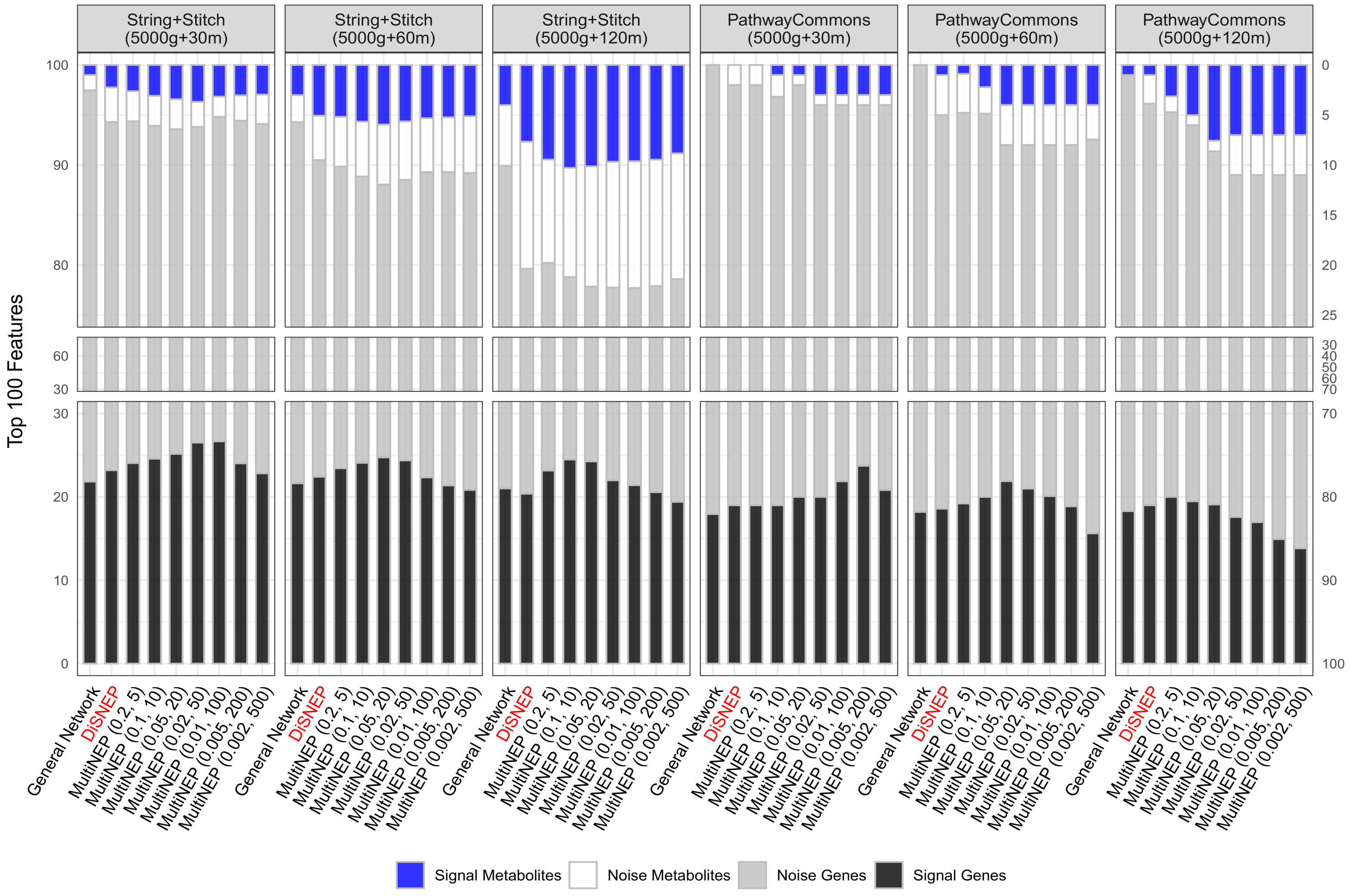

Figure S4: Simulation results to determine optimal values of λ_g and λ_m when disease profiles have different numbers of genes and metabolites. Displayed are average numbers of signal genes (black), signal metabolites (blue), noise genes (grey), and noise metabolites (white) out of top ranked 100 prioritized combined features across 100 simulations when S^0 is from STRING & STITCH (panel 1-3) or PathwayCommons (panel 4-6) containing 5,000 genes and 30 (panel 1, 4), 60 (panel 2, 5), or 120 (panel 3, 6) metabolites. Correlations between signal genes and signal metabolites were set at $\rho = 0.5$. λ_g = 0.2, 0.1, 0.05, 0.02, 0.01, 0.005, 0.002 and λ_m = 5, 10, 20, 50, 100, 200, 500 for MultiNEP (λ_g, λ_m). Models prioritized the most number of true signal genes and metabolites under each simulation setting are circled in red.

References

B. S. Carvalho and R. A. Irizarry. A framework for oligonucleotide microarray preprocessing. *Bioinformatics*, 26(19):2363–2367, Aug. 2010. doi:10.1093/bioinformatics/btq431.

E. G. Cerami, B. E. Gross, E. Demir, I. Rodchenkov, O. Babur, N. Anwar, N. Schultz, G. D. Bader, and C. Sander. Pathway commons, a web resource for biological pathway data. *Nucleic Acids Research*, 39 (Database):D685–D690, Nov. 2010. doi:10.1093/nar/gkq1039.

K. Chetnik, E. Benedetti, D. P. Gomari, A. Schweickart, R. Batra, M. Buyukozkan, Z. Wang, M. Arnold, J. Zierer, K. Suhre, and J. Krumsiek. ttmaplet/tt: an extensible r toolbox for modular and reproducible metabolomics pipelines. *Bioinformatics*, 38(4):1168–1170, Oct. 2021. doi:10.1093/bioinformatics/btab741.

F. Dieterle, A. Ross, G. Schlotterbeck, and H. Senn. Probabilistic quotient normalization as robust method to account for dilution of complex biological mixtures. application in sup1/suph NMR metabonomics. *Analytical Chemistry*, 78(13):4281–4290, May 2006. doi:10.1021/ac051632c.

K. T. Do, S. Wahl, J. Raffler, S. Molnos, M. Laimighofer, J. Adamski, K. Suhre, K. Strauch, A. Peters, C. Gieger, C. Langenberg, I. D. Stewart, F. J. Theis, H. Grallert, G. Kastenmüller, and J. Krumsiek. Characterization of missing values in untargeted MS-based metabolomics data and evaluation of missing data handling strategies. *Metabolomics*, 14(10), Sept. 2018. doi:10.1007/s11306-018-1420-2.

R. A. Irizarry. Summaries of affymetrix GeneChip probe level data. *Nucleic Acids Research*, 31(4):15e–15, Feb. 2003. doi:10.1093/nar/gng015.

K. L. Penney, S. Tyekucheva, J. Rosenthal, H. E. Fandy, R. Carelli, S. Borgstein, G. Zadra, G. N. Fanelli, L. Stefanizzi, F. Giunchi, M. Pomerantz, S. Peisch, H. Coulson, R. Lis, A. S. Kibel, M. Fiorentino, R. Umeton, and M. Loda. Metabolomics of prostate cancer gleason score in tumor tissue and serum. *Molecular Cancer Research*, 19(3):475–484, Mar. 2021. doi:10.1158/1541-7786.mcr-20-0548.

M. E. Ritchie, B. Phipson, D. Wu, Y. Hu, C. W. Law, W. Shi, and G. K. Smyth. limma powers differential expression analyses for RNA-sequencing and microarray studies. *Nucleic Acids Research*, 43(7):e47–e47, Jan. 2015. doi:10.1093/nar/gkv007.

A. Terunuma, N. Putluri, P. Mishra, E. A. Mathé, T. H. Dorsey, M. Yi, T. A. Wallace, H. J. Issaq, M. Zhou, J. K. Killian, H. S. Stevenson, E. D. Karoly, K. Chan, S. Samanta, D. Prieto, T. Y. Hsu, S. J. Kurley, V. Putluri, R. Sonavane, D. C. Edelman, J. Wulff, A. M. Starks, Y. Yang, R. A. Kittles, H. G. Yfantis, D. H. Lee, O. B. Ioffe, R. Schiff, R. M. Stephens, P. S. Meltzer, T. D. Veenstra, T. F. Westbrook, A. Sreekumar, and S. Ambs. MYC-driven accumulation of 2-hydroxyglutarate is associated with breast cancer prognosis. *Journal of Clinical Investigation*, 124(1):398–412, Dec. 2013. doi:10.1172/jci71180.
